# A Rare Case of Ipilimumab-induced Reversible Hypophysitis and Permanent Primary Hypothyroidism

**DOI:** 10.7759/cureus.5001

**Published:** 2019-06-25

**Authors:** Amani Erra, Bibek Singh Pannu, Sabah Patel, Faisal Qureshi, Mohanad Soliman

**Affiliations:** 1 Internal Medicine, Myeloma Institute, University of Arkansas for Medical Sciences, College of Medicine, Little Rock, USA; 2 Internal Medicine, Presence Saint Joseph Hospital, Chicago, USA; 3 Internal Medicine, Endocrinology, AMITA Health Saint Joseph Hospital Chicago, Chicago, USA; 4 Internal Medicine, University of Kentucky, Lexington, USA

**Keywords:** ipilimumab, immune related adverse events, hypophysitis, melanoma

## Abstract

Ipilimumab is a monoclonal antibody targeting the cytotoxic T-lymphocyte antigen-4 receptor, which was originally approved for the treatment of metastatic melanoma. It is the first immune checkpoint inhibitor to enter clinical practice. Immune toxicity due to ipilimumab causing colitis, hepatitis, and dermatitis are well-described in literature. We report a case of hypophysitis resolving with corticosteroid treatment, following which the patient developed long-term primary thyroid impairment. This highlights the importance of vigilance for rarer immune-related toxicities as clinical utilization of ipilimumab becomes more widespread.

## Introduction

Check points and gene repair are a crucial part of natural immunity. Cytotoxic T lymphocytes - 4 (CTLA-4) receptor is normally expressed by activated T cells and functions as an inhibitory regulator of cytotoxic T cells, thereby causing a downregulation of the immune response. Ipilimumab, a human monoclonal antibody against CTLA-4, binds to the receptor and blocks the inhibitory signal, thus antagonizing the immune system tolerance to tumor. Subsequently, multiple trials have been conducted in different cancers showing a promising response to ipilimumab. With increasing use, there are also an increasing number of side effects that have been identified, in upto more than half of the patients. Up regulation of immune system can in turn target host cells and manifest as various toxicities [1]. We present a case of a 63-year-old male with stage 3 melanoma; he underwent tumor resection followed by treatment with ipilimumab. He developed nonspecific symptoms of headache and malaise, which were later linked to hypophysitis, and related side effects with long term endocrine toxicities involving the thyroid and adrenal glands. He required treatment with steroids and hormone replacement for persistent symptoms and pathology. Here, we also discuss the etiology of immune-related adverse events (IRAE) due to ipilimumab, long-term sequelae, treatment strategies, and outcomes.

## Case presentation

**Figure 1:**
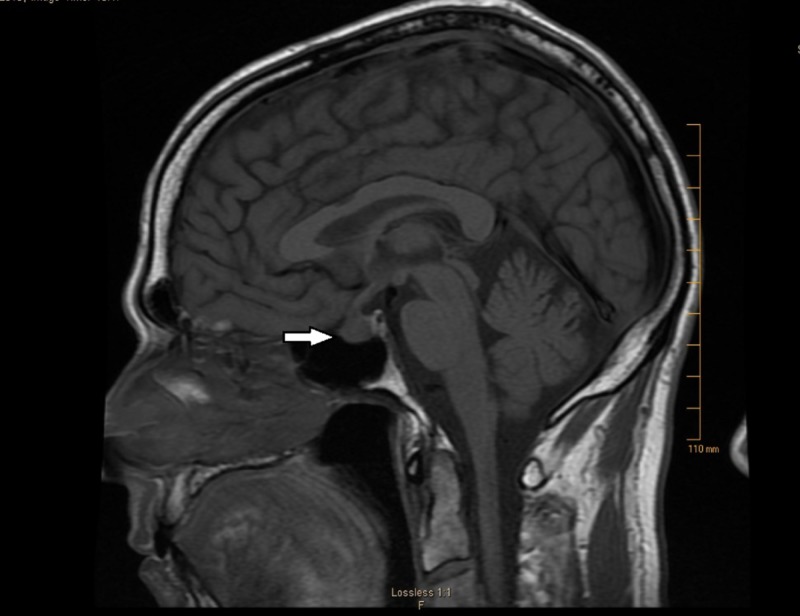
Sagittal section, T1 weighted image showing pituitary enlargement consistent with the diagnosis of hypophysitis

A 63-year-old male with a past medical history of hypertension, erectile dysfunction, and hyperlipidemia was diagnosed with stage 3 melanoma of the scalp with positive right retro-auricular lymph nodes. He underwent surgical excision of the tumor and biopsy revealing a Breslow depth of 4.5 mm with Clark level 5. Magnetic resonance imaging (MRI) of the brain was negative for intracranial metastatic disease at the time. He was then started on ipilimumab 10 mg/kg every three weeks. After finishing four cycles of ipilimumab, he reported recurrent left-sided retro-orbital headache associated with photosensitivity, nasal congestion, and clear discharge. He denied any nausea, vomiting, weakness, dizziness, gynecomastia, or vision changes. Physical examination was essentially unremarkable with no visual field abnormality. He was initially treated for possible sinusitis with decongestants and antibiotics. Upon non-resolution of his symptoms, a repeat MRI brain with contrast was performed, which revealed an increase in the size of pituitary gland from 0.8 x 0.4 cm to 1.1 x 0.8 cm as noted in the image (Figure 1).

The constellation of brain imaging findings, ongoing symptoms along with current immunotherapy were all suggestive of ipilimumab-related hypophysitis (IRH). Empiric prednisone at 1 mg/kg was started after obtaining early morning adrenocorticotropic hormone (ACTH) and cortisol levels. His laboratory findings were significant for a low cortisol of 0.8 mcg/dL (7-25 mcg/dL), an inappropriately low normal ACTH of 21 Pg/ml (7-69 Pg/ml) , with normal potassium of 4.2 mmol/L (3.5-5.1 mmol/L), and a low sodium of 131 mmol/L (133-144 mmol/L), which was interestingly suggestive of possible underlying secondary adrenal insufficiency. However, in the absence of workup with cosyntropin test, a definitive diagnosis could not be established. His serum follicle-stimulating hormone (FSH), luteinizing hormone (LH), and testosterone levels were normal. Other labs were notable for prolactin 9 ng/dl (normal <20 ng/dl) and IGF 53 ng/ml (33-220 ng/ml). Ipilimumab was discontinued at this point. He was initially treated for a period of three months and noted significant symptom resolution; his serum sodium levels improved to normal.

Prednisone was decreased to 50 mg daily and subsequently tapered to 10 mg daily. Upon prednisone dose reduction, the patient experienced a relapse of his headache. A slow prednisone taper regimen over a period of eight months was started. On follow-up, symptoms of headache had resolved, and a repeat MRI of the brain six months later showed a reduction and normalization of pituitary size. The patient was also started on levothyroxine 100 mcg for secondary hypothyroidism, which was discontinued two months later as his serum thyroid stimulating hormone (TSH) and free thyroxine 4 (FT4) levels normalized. Four months later, during regular follow-up his TSH was 12.4 (0.4 - 4.5 mIU/L) demonstrating pituitary recovery but FT4 was 0.81 (5 - 16 mg/dL), suggesting the development of primary hypothyroidism. He was re-started on levothyroxine 25 mcg and repeat TSH was 11.9 mIU/ml and FT4 was 5 mg/dl, and his levothyroxine was continued. Further workup was negative for thyroid peroxidase and thyroglobulin antibodies.

Ipilimumab was not re-started because of the significant hypophysitis. His headache resolved and his thyroid function improved. Attempts at weaning off prednisone were unsuccessful resulting in return of symptoms including severe headaches. The patient was hence continued on 2.5 mg of prednisone once daily as a maintenance dose. He effectively received four cycles of ipilimumab, and the melanoma has been in remission since then. Although the original plan was to continue ipilimumab with 12 weekly dosing for three years, given the severity of the side effects, the drug was not restarted. He has regular follow-up for testing including testing for serum TSH, cortisol, and serum sodium.

## Discussion

Ipilimumab was first approved by the Food and Drug Administration (FDA) in March 2011 for the treatment of stage III and IV melanoma, with a dramatic improvement in prognosis and overall survival in such patient populations [1]. Immune-related side effects have been reported during induction and re-induction phases. Endocrine-related side effects such as nausea, fatigue, amenorrhea, hypotension, hyponatremia, hypoglycemia or eosinophilia are generally mild and late to be diagnosed, and these are seen in up to 60% of the patients. Grade 3 or 4 events can be seen in up to 10%-15% of patients [2].

Amongst the ipilimumab-related endocrinopathies, pituitary dysfunction (hypophysitis) is identified in 9%-13% of patients; other less common endocrine toxicities like primary hypothyroidism or hyperthyroidism and primary adrenal insufficiency are reported as well [3]. Interestingly, hypophysitis is more common in males as compared to females. Its onset ranges from 4-16 weeks after initiating the treatment and may result in deficiencies in one or more anterior pituitary gland hormones [3, 4].

Immune-related hypophysitis (IRH) is more prominent after the third cycle of immunotherapy and with higher dosage. It is proposed that this toxicity due to ipilimumab involves antibodies targeting TSH- and FSH-secreting cells in the pituitary gland, which stimulate complement activation and result in cellular injury [5].

Hypophysitis typically manifests with a headache, visual field defects and rarely diplopia. Although brain imaging is not required to diagnose it, certain imaging characteristics on brain MRI such as homogenous enhancement of the pituitary, diffuse symmetric gland enlargement, midline stalk thickening, and the absence of a posterior pituitary bright spot correlate with hypophysitis [6].

Treatment of IRH starts with discontinuation of ipilimumab. There is conflicting evidence about the role of corticosteroid as a symptomatic treatment. Some authors suggest that steroids do not reverse hypopituitarism and that therapy should be targeted, instead, towards treating secondary hormonal abnormalities [7]. The expected median time for resolution of IRH is between 3.1 to 6.3 weeks [1].

Ipilimumab-related primary hypothyroidism presents in 5.6% (5.2%-5.9%) as early as five months up to three years. Fatigue is the most common presenting complaint in these patients, and it improves after thyroid hormone replacement [3]. In a comprehensive retrospective review, the overall incidence of IRH and primary hypothyroidism were 8% and 6%, respectively [8]. Secondary adrenal insufficiency as a result of IRH is mostly permanent [7]. Primary adrenal involvement has been rarely reported [7]. Immune-related adverse events other than endocrinopathies have also been reported with ipilimumab; for example skin rash and pruritus in 50% of the patients, and diarrhea that is dose-dependent in 30% of the patients. Pneumonitis, a diagnosis of exclusion, is an uncommon yet potentially fatal presentation of immune-related adverse events (IRAEs) [9].

The question of rechallenging with immune checkpoint inhibitors in patients with a history of IRAEs is still being widely studied. There is some evidence emerging about safe retreatment with ipilimumab, but further studies are still needed [10].

## Conclusions

Ipilimumab has been a popular and widely utilized agent in the treatment of melanoma. It is imperative that physicians be aware of hypophysitis as an important endocrine immune-related adverse event. This usually presents with non-specific symptoms like fatigue and headache, which can be easily dismissed as a minor ailment or may be attributed to other more common comorbidities. Hence, a high suspicion remains the key to early diagnosis. Treatment entails use of glucocorticoids and hormonal replacement as appropriate along with frequent laboratory testing and regular follow-up.
